# Changes in the Transcriptome of the Human Endometrial Ishikawa Cancer Cell Line Induced by Estrogen, Progesterone, Tamoxifen, and Mifepristone (RU486) as Detected by RNA-Sequencing

**DOI:** 10.1371/journal.pone.0068907

**Published:** 2013-07-16

**Authors:** Karin Tamm-Rosenstein, Jaak Simm, Marina Suhorutshenko, Andres Salumets, Madis Metsis

**Affiliations:** 1 Centre for Biology of Integrated Systems, Tallinn University of Technology, Tallinn, Estonia; 2 Competence Centre on Reproductive Medicine and Biology, Tartu, Estonia; 3 Nova Vita Clinic, Tallinn, Estonia; 4 Department of Obstetrics and Gynaecology, University of Tartu, Tartu, Estonia; 5 Institute of Biomedicine, University of Tartu, Tartu, Estonia; 6 Institute of Mathematics and Natural Sciences, Tallinn University, Tallinn, Estonia; Baylor college of Medicine, United States of America

## Abstract

**Background:**

Estrogen (E2) and progesterone (P4) are key players in the maturation of the human endometrium. The corresponding steroid hormone modulators, tamoxifen (TAM) and mifepristone (RU486) are widely used in breast cancer therapy and for contraception purposes, respectively.

**Methodology/Principal findings:**

Gene expression profiling of the human endometrial Ishikawa cancer cell line treated with E2 and P4 for 3 h and 12 h, and TAM and RU486 for 12 h, was performed using RNA-sequencing. High levels of mRNA were detected for genes, including *PSAP, ATP5G2, ATP5H*, and *GNB2L1* following E2 or P4 treatment. A total of 82 biomarkers for endometrial biology were identified among E2 induced genes, and 93 among P4 responsive genes. Identified biomarkers included: *EZH2, MDK, MUC1, SLIT2,* and *IL6ST*, which are genes previously associated with endometrial receptivity. Moreover, 98.8% and 98.6% of E2 and P4 responsive genes in Ishikawa cells, respectively, were also detected in two human mid-secretory endometrial biopsy samples. TAM treatment exhibited both antagonistic and agonistic effects of E2, and also regulated a subset of genes independently. The cell cycle regulator cyclin D1 (*CCND1*) showed significant up-regulation following treatment with TAM. RU486 did not appear to act as a pure antagonist of P4 and a functional analysis of RU486 response identified genes related to adhesion and apoptosis, including down-regulated genes associated with cell-cell contacts and adhesion as *CTNND1, JUP, CDH2, IQGAP1,* and *COL2A1.*

**Conclusions:**

Significant changes in gene expression by the Ishikawa cell line were detected after treatments with E2, P4, TAM, and RU486. These transcriptome data provide valuable insight into potential biomarkers related to endometrial receptivity, and also facilitate an understanding of the molecular changes that take place in the endometrium in the early stages of breast cancer treatment and contraception usage.

## Introduction

The ovarian steroid hormones, estrogen (E2) and progesterone (P4), play crucial roles in regulating normal functions of the human endometrium. For example, during a normal menstrual cycle, proliferation, differentiation, and degeneration of the endometrium occur in response to varying E2 and P4 levels. In the proliferative phase, E2 stimulates the proliferation of epithelial cells and stromal components of the endometrium. In the secretory phase, P4 modulates glandular differentiation and an inhibition of estrogen-mediated proliferation [1]. It is during the mid-secretory phase that the endometrium achieves a phenotype compatible with successful embryo implantation.

A better understanding of the biology and functioning of the human endometrium is vital to improving our knowledge about female infertility, and for the design of treatments for these conditions. Correspondingly, a search for markers of endometrial receptivity and novel approaches to improve implantation rates during infertility treatments have been conducted. In recent years, numerous studies involving microarray expression analysis have identified a wide range of genes up- or down-regulated in the human endometrium during the time of embryo implantation [2]–[10]. Each study identified many candidate genes believed to be critical to the embryo implantation process. However, few genes were consistently reported.

The genomic activities of E2 and P4 are mainly mediated by nuclear receptors. When E2 or P4 are bound to their receptors, they can bind response elements in DNA with high affinity and regulate the transcription of target genes. In humans, there are two types of estrogen receptors, ERα and ERβ, and these are encoded by separate genes [11], [12]. ERα and ERβ are expressed in all endometrial cell types throughout the entire menstrual cycle, and undergo changes in expression and activity. For example, ERα and ERβ are expressed at higher levels during the proliferative phase, yet exhibit lower activity during the secretory phase when they are subject to the suppressive effects of P4. It is after the proliferative phase that P4 mediates E2-based priming of the endometrium towards a state of receptivity.

In contrast with ERα and ERβ, the progesterone receptors (PRs), PRA and PRB, are encoded by the same gene (*PGR*), yet are transcribed from different promoters. As a result, PRB includes an additional 164 amino acids at its N-terminus [13], [14]. Both isoforms are expressed in the stroma and epithelium of the endometrium during the proliferative phase. However, the expression of both receptors decreases sharply in the epithelium during the early to mid-secretory phase [15]. Endometrial receptivity appears to be tightly associated with the down-regulation of epithelial PRs, while the stroma only maintains expression of PRA during the secretory phase [16]. Expression of PR genes in the endometrial glandular epithelium is controlled by E2 and P4, with E2 inducing PR synthesis and P4 down-regulating expression of its own receptor [1].

Selective ER modulators (SERMs) have the ability to interact with ERs as agonists or antagonists depending on the target tissue and to modulate signal transduction pathways of E2-responsive genes [17]. For example, tamoxifen (TAM) binds with high affinity to ERs, thereby blocking the action of native E2. Due to its antagonistic activity of E2, TAM has been widely used in breast cancer therapy. However, one of the most troublesome side effects of breast cancer treatments with TAM appears to be its proliferative effect on the endometrium [18], [19]. For example, endometrial pathologies associated with TAM treatments include hyperplasia, polyps, carcinomas, and sarcomas [20]. Similar to SERMs, selective progesterone receptor modulators (SPRMs) have been developed to antagonize processes activated by P4. Mifepristone (RU486) is a P4 antagonist that competes with endogenous P4 for receptor binding [21] and is used to end an early pregnancy. RU486 also exhibits a 2-to-10-fold higher affinity towards PRs compared to P4 [22].

The exact basis for the differential, tissue-specific signalling of E2 and P4 is still not fully understood, and a better understanding of E2 and P4 actions is needed to evaluate their roles in regulating endometrial gene expression. In this study, the human uterine-derived epithelial cancer cell line, Ishikawa, was used. It is one of the most well-characterized human endometrial cell lines currently available. Ishikawa cells were derived from a well-differentiated adenocarcinoma of the human endometrial epithelium that expressed functional steroid receptors for E2 and P4 [23]–[25]. As a result, this cell lines represents an ideal model for studying the response of the endometrial epithelium to E2 and P4. In this study, high-throughput RNA-sequencing (RNA-Seq) was applied to studies of E2- and P4-dependent transcriptomes in an endometrial context. The steroid hormone receptor modulators, TAM and RU486, were also used to study receptor-dependent signal transduction, and to evaluate agonistic or antagonistic activity in the Ishikawa cell line. Finally, the significant E2- and P4-dependent genes identified in the Ishikawa cell line were assayed in endometrial biopsy samples collected at the receptive mid-secretory phase.

## Materials and Methods

### Cell Culture

The Ishikawa cell line was provided by Prof. Anneli Stavreus-Evers (Uppsala University, Sweden). Cells were grown in DMEM medium (PAA, Pasching, Austria), supplemented with 5% fetal bovine serum (FBS; PAA) and 1% penicillin/streptomycin (PAA), at 37°C and 5% CO_2_. For hormonal treatments, E2 (β-Estradiol) or P4 (4-Pregnene-3,20-dione) were added to the culture media to a final concentration of 10^−8 ^M. The steroid hormone modulators, tamoxifen (TAM, 4-hydroxytamoxifen) and mifepristone (RU486), were added to culture media to a final concentration of 1 µM. All hormones and modulators were ordered from Sigma-Aldrich (Schnelldorf, Germany), with E2 and P4 dissolved in dimethylsulfoxide (DMSO) and TAM and RU486 in ethanol (EtOH). Control samples were treated with vehicle only. For cultures with hormone supplements, dextran-coated, charcoal-treated FBS and media without phenol red were used 48 h prior to experiments to avoid possible hormone-like activity of phenol red.

### RNA Extraction

Total RNA was extracted from untreated cells and cells after 3 h and 12 h of hormone treatment using RNeasy Mini Kits (Qiagen, Valencia, USA). Endometrial biopsies were collected from two patients (ages, 34 and 38 years) with unexplained infertility treated at the Nova Vita Clinic. Biopsies were collected on days LH+7 to LH+9 according to urine ovulation tests. Tissue samples were homogenized with Tissue lyzer (Qiagen) and total RNA was extracted from previously formalin-fixed (3.7%) endometrial biopsies using an RNeasy FFPE kit (Qiagen) according to the manufacturer’s instructions. The study was carried out on accordance with the local ethical standards and was approved by the Ethics Review Committee on Human Research of the University of Tartu, with written consent obtained from both study participants.

### RNA Library Preparation

RNA libraries for cell line and endometrial samples were prepared using an Illumina TruSeq RNA Sample Prep Kit (FC-122-1001, Illumina, San Diego, USA) according to the manufacturer’s instructions. For cell line samples, mRNA was purified using polyA selection, and was subsequently fragmented chemically. For tissue samples, total RNA was collected without polyA selection and the fragmentation step was shortened based on the previous formalin treatment of the samples. In all samples, RNAs were converted into single-stranded cDNAs using random hexamer priming. Multiplexing with different adapter indexes was performed and the quality of the resulting library was checked using a Bioanalyzer (Agilent, Waldbronn, Germany).

### RNA-Seq and Data Analysis

Single-end (SE) sequencing of 75 bp was performed using an Illumina Genome Analyzer II (Illumina, San Diego, USA). Bowtie programming was used to provide an initial alignment of sequences to human genome 19 (Hg19), with default settings used to find only perfect matches. Sequenced fragments were aligned to the *H. Sapiens* reference genome (Hg19) provided by University of California Santa Cruz (UCSC) Genome browser using a TopHat v1.2.0 algorithm with default settings [26]. The aligned reads were subsequently processed into transcripts using Cufflinks v1.1.0 [27], with abundances estimated and analysed to examine differential expression patterns between cell line samples. Cufflinks constructed a minimum set of transcripts to best describe the reads in the dataset. The Benjamin-Hochberg correction for multiple testing was applied to the P values of significant genes with a false discovery rate (FDR) value of 0.05. Normalized RNA-Seq fragment counts indicating the relative abundances of the transcripts were used. Abundances were reported in units of FPKM (e.g., Fragments Per Kilobase of transcript per Million of fragments mapped). The output files of Cufflinks were analysed with Cuffcompare along with the reference from the UCSC Table Browser (Homo sapiens GRCh37/Hg19) [28]. Cuffcompare classifies each transcript as known or novel. Cuffdiff re-estimates the abundance of transcripts listed by Cuffcompare and tests for differential expression between the selected experiments. If one of the experiments (either control or treatment) had 0 FPKM, the log change became infinite. We expressed the log change in these cases as +14 for up-regulation and -14 for down-regulation.

### Functional Analysis

For the functional classification of genes that exhibited significant differential expression profiles in response to different steroid hormone and their analogue treatments, Ingenuity Pathway Analysis (IPA) 9.0 software (Ingenuity Systems) was used. The IPA transcription factor module was used to predict the gene expression changes detected regarding to potential bindings of ERs and PRs. In addition, IPA biomarker analysis filters identified potential biomarkers in selected tissues.

### Data Visualization

R statistics software (version 2.14.0) (http://www.R-project.org/) was used to process and visualize the results from Cufflinks analyses. Calculation of general statistics, including common and unique counts of significantly affected genes, were performed in R using a custom script. For heatmap visualizations, the R package gplots (version 2.10.1) (http://CRAN.R-project.org/package=gplots) was used. In addition, differences in the FPKM values of the treated samples versus the non-treated samples were calculated in the heatmaps. The largest absolute FPKM difference for each gene was identified, and was used to normalize FPKM data for each gene. Thus, the resulting values lie between −1 and 1, and a value of 0 corresponds to an absence of change compared to the non-treated sample. Based on these normalized expression values, genes were positioned in the heatmap by hierarchical clustering.

## Results

### The Transcriptome of the Ishikawa Cell Line Before and After Treatment with E2, P4, and Respective Modulators

PolyA-selected RNA from the human endometrial cell line, Ishikawa, was subjected to SE-sequencing with 75 basepair long reads. Reference measurements for each sample were then made based on the 8–11×10^6^ reads that were obtained. The goal of this sequencing effort was to provide an overall gene expression profile of the Ishikawa cell line in order to identify changes in gene expression that occur during the early response of this cell line to steroid hormones and their modulators. Altogether, seven samples were analysed, and these included non-treated cells, cells treated with E2 or P4 for 3 and 12 h, and cells treated with TAM or RU486 modulators for 12 h. The majority of reads from each sample (e.g., >70%) were successfully aligned to the human genome version 19 (Hg19). Statistical values of these alignments and the number of genes identified, including both known and unknown genes, are listed in Table 1. The relative abundances of fragments were calculated using Cufflinks, and were reported in units of FPKMs in order to describe expressed genes (e.g., fragments) observed from RNA-Seq experiments. In Table 1, the number of genes with different FPKM abundances, as well as the numbers of genes which exhibited significant changes in expression following hormone/modulator treatment, were compared with non-treated cells. In addition, the most responsive genes identified from the Ishikawa cell line were compared with human endometrium biopsy samples (n = 2) collected during the time of embryo implantation. A complete list of the expressed genes identified and their FPKM values are available in Table S1.

**Table 1:** RNA-Seq statistics of E2, P4, TAM, and RU486 treated and non-treated Ishikawa cells.

		Ishikawa E2 & TAM			Ishikawa P4 & RU486		
	Non-treated	3 h E2	12 h E2	12 h TAM	3 h P4	12 h P4	12 h RU486
**Reads aligned to Tophat**	10 465 431	9 967 453	8 093 476	10 912 784	10 628 213	11 058 071	10 983 639
**Total genes**	16813	16874	16784	16834	16951	16934	16840
**% unknown genes**	29.07%	29.09%	28.89%	28.86%	29.43%	29.24%	29.06%
**% known genes**	70.93%	70.91%	71.11%	71.14%	70.57%	70.76%	70.94%
**FPKM 0**–**10**	4577	4361	4226	4611	4437	4406	4607
**FPKM 10**–**100**	6487	6701	6894	6486	6653	6722	6495
**FPKM 100**–**1000**	820	859	767	831	831	807	804
**FPKM >1000**	41	44	48	47	41	47	40
**Significant genes** *		1084	1121	1013	1082	1097	546
**% endometrium (n = 2)** **		98.6%	99.2%	98.6%	98.2%	99.0%	98.5%

* Significant genes (5% FDR) are counted from known genes and compared to non-treated cells.

** E2 and P4 significant genes present in human endometrium during the time of embryo implantation.

One of the advantages of a RNA-Seq analysis is the ability to detect relatively high expression levels of genes. A subset of genes from the Ishikawa cell line had FPKM values that were greater than 1000 after hormone/modulator treatments (Table 2). These included genes encoding prosaposin (*PSAP*), ATP synthases, *ATP5G2* and *ATP5H*, and guanine nucleotide binding protein (*GNB2L1*). These genes were very highly expressed in response to E2 or P4 treatment. The expressions of *ATP5G2*, *ATP5H* and *GNB2L1* have not been shown to be related to endometrium before. Alternatively, genes encoding the S100 calcium binding proteins A2 (*S100A2*) and A6 (*S100A6*), heat shock protein 90 kDa alpha (*HSP90AA1*), and HSPA8, as well as pyruvate kinase in muscle (*PKM2*), exhibited high levels of expression 12 h after treatment with TAM. Among these genes only the expression of *S100A2* has been related to TAM treatment in breast cancer tissue but not in endometrium [29]. In addition, the gene for ferritin light polypeptide (*FTL*), also not detected in endometrium in former studies, was found to be highly expressed 12 h after treatment with E2, P4, and TAM (Table 2).

**Table 2:** Selection of genes with FPKM >1000 after hormone/modulator treatments.

Gene name	Description	Non-treated	3 h E2	12 h E2	12 h TAM	3 h P4	12 h P4	12 h RU486
S100A2	S100 calcium binding protein A2	178.9	145.2	75.3	1469.4*	182.2	137.6	105.8
S100A6	S100 calcium binding protein A6	451.9	421.9	351	1141.6*	414.4	330.9	492
PSAP	prosaposin	875.8	988.4	1021.6*	710.2	933.2	962.8	771.1
HSPA8	heat shock 70 kDa protein 8	840.8	802.4	880.6	1496.2*	702.5	925.8	982.1
ATP5G2	ATP synthase, H+ transporting,mitochondrial Fo complex, subunit C2	961.8	1055.3*	1100.2*	858.3	1041.1*	1232.6*	779.1
HSP90AA1	heat shock protein 90 kDa alpha	733.3	742.7	815	1277.4*	760.9	891	884.7
PKM2	pyruvate kinase, muscle	642.5	565.3	641.8	1019.0*	538.2	597.6	653.3
ATP5H	ATP synthase, H+ transporting,mitochondrial Fo complex, subunit d	974.9	1054.7*	914.1	910.7	1178.3*	907.9	890
FTL	ferritin, light polypeptide	761	687	1027.5*	1130.9*	726.7	1056.5*	989.7
GNB2L1	guanine nucleotide binding protein	851.3	948.5	1027.8*	778.9	891.9	1006.5*	748.8

* FPKM abundance >1000.

### Significant Gene Expression Changes in the Ishikawa Cell Line After E2 and P4 Treatments

Relative mRNA expression levels (in units of FPKM) for E2- and P4-treated cells were compared with non-treated cells using Cuffdiff software (version 1.1.0) and a 5% FDR. The number of genes that exhibited significant changes in expression after respective treatments are listed in Table 1. In addition, only known genes were included in subsequent analyses of gene expression data.

A total of 1691 known genes (Table S2) were found to be significantly affected in Ishikawa cells following treatments with E2 for 3 h (n = 1084) and 12 h (n = 1121) compared to non-treated cells. Of those genes, 614 were significantly up-regulated, and 470 were significantly down-regulated after 3 h of E2 treatment. When treatment with E2 was extended to 12 h, induction of 715 genes, and suppression of 406 genes, was detected.

In majority of genes 12 h TAM treatment showed antagonistic activity of E2. Twelve hours of treatment with TAM resulted in low or undetectable levels of mRNA for 654 (91.5%) genes of the 715 genes that exhibited higher mRNA levels following treatment with E2 for 12 h. An additional 406 genes were found to be down-regulated following treatment with E2 for 12 h, and 75.1% (n = 305) of these genes exhibited only minor changes in gene activity, or were associated with an absence of regulation, 12 h after treatment with TAM.

Based on the data obtained, TAM did not act as a pure antagonist of E2 in the Ishikawa cell line. For example, of the 715 genes up-regulated after treatment with E2 for 12 h, 61 (8.5%) were also significantly up-regulated following treatment with TAM. In addition, among the 406 genes that were found to be down-regulated following treatment with E2 for 12 h, 101 (24.9%) were similarly down-regulated following treatment with TAM. In combination, these data demonstrate that TAM is both antagonistic and agonistic for E2 in the Ishikawa cell line (Figure S1).

Following treatment with P4, a total of 1692 known genes exhibited significant differences in expression (Table S3). Of these genes, 1082 exhibited changes after 3 h of treatment, and 1097 were detected following 12 h of treatment, both compared to non-treated Ishikawa cells. Of these genes, 592 (54.7%) were significantly up-regulated, and 490 (45.3%) were down-regulated 3 h after treatment with P4, while 631 (57.5%) were up-regulated and 466 (42.5%) were down-regulated 12 h after treatment with P4.

When Ishikawa cells were treated with RU486 for 12 h, low or undetectable levels of mRNA were detected for 84.0% (n = 530) of genes which were significantly up-regulated following treatment with P4 for 12 h (n = 631). Another 466 genes were found to be down-regulated following treatment with P4 for 12 h, and of these, 88.2% (n = 411) exhibited minor down-regulation, or showed no regulation, following treatment with RU486 for 12 h.

Of the 631 genes that were up-regulated in response to 12 h P4 treatment, 101 (16.0%) of these genes were also significantly up-regulated following treatment with RU486. Alternatively, of the 466 genes down-regulated in response to treatment with P4 for 12 h, 55 (11.8%) exhibited a similar down-regulation following treatment with RU486. Moreover, similar to TAM, RU486 exhibited both agonistic and antagonistic activity of P4 in Ishikawa cells (Figure S2).

Of the 1691 genes significantly responsive to E2, and the 1692 genes significantly responsive to P4, 1051 were common to both groups, suggesting that they are regulated by both hormones. Relative majority of the genes identified with significant changes after E2 and P4 treatment, have not been mentioned in endometrial context before.

### Potential ER and PR Targets Among the E2 and P4 Significant Genes Identified

An IPA analysis of genes found to be responsive to E2 revealed 20 potential target genes for the E2 receptor, ERα (*ESR1*), based on a database of experimentally observed receptor interactions. For example, *ABCA3, CELSR2, CYP1A1, DDX17, EFEMP1, ENO1, FOSL2, GREB1, KCNK6, MAPK12, MYC, PDCD4, PGR, SHANK3, TGFA,* and *TPM1* were significantly up-regulated following treatment with E2 for 12 h. Conversely, *CCNG2, KCTD6, LDLR,* and *PRLR* were down-regulated. Of these gene products, PGR and MAPK12 participate in glucocorticoid receptor signalling, whereas MYC and CYP1A1 are involved in aryl hydrocarbon receptor (AHR) signalling (Figure S3).

After 12 h of treatment with P4, expression of *CDKN1C, F3, FKBP5, GAS6, IL1R1, NQO2, PFKP, TSC22D3* and *PGR* were found to be up-regulated, while *EZR*, *ACSL1, AKAP13, CCNB2, GLUL, MYCN, PPIF,* and *SNTB2* were found to be down-regulated. Of these, CDKN1C, FKBP5, and PGR contribute to glucocorticoid receptor signalling, while ACSL1 and PFKP have roles in gluconeogenesis (Figure S4).

### Biomarker Analysis of E2 and P4 Significant Genes from the Ishikawa Cell Line

Next, potential biomarkers among the E2 (n = 1691) and P4 (n = 1692) responsive genes were examined. Only molecules previously detected in the human endometrium were considered for the IPA biomarker analysis performed, and molecules were further filtered to include those related to reproductive system diseases or endocrine system disorders.

IPA biomarker analysis identified 82 potential biomarkers among E2 significant genes and 93 potential biomarkers among P4 significant genes. There were 62 potential biomarker molecules common to both groups. A complete list of biomarkers expressed in the human uterus is compared with biomarkers identified in Ishikawa cells 3 h and 12 h after E2, P4, and respective modulator treatments (Table S4). Selection of unique E2 and P4 dependent biomarkers that have been related to endometrial receptivity and embryo implantation are listed in Table 3. Regarding the former, these included the following genes related to the endometrium and embryo implantation: *MUC1, EZH2, HMGCR, MDK, PRDM2, PXN,* and *SLIT2* (Table 3). For genes common to both E2 and P4 responsive genes, potential biomarkers were identified that were related to endometrial receptivity or endometriosis, and these included: *ARG2, ANXA1, AR, BMPR2, CDKN1C, CXCL16, EGFR, FGFR1, HMGA1, IGFR2, IL1R1, JAG1, let-7, MCAM, NCOA3, NOTCH1, PDCD4, PGR, RACGAP1, TMSB10,* and *TNC* (Table S4).

**Table 3:** Selection of biomarkers related to reproductive system diseases among E2 and P4 significant genes in Ishikawa cell line.

E2 UniqueSymbol	Entrez Gene Name	E2 3 hLog Ratio	E2 12 hLog Ratio	TAM 12 hLog Ratio
EZH2	enhancer of zeste homolog 2 (Drosophila)	−0.184	−14	−0.089
HMGCR	3-hydroxy-3-methylglutaryl-CoA reductase	−0.164	−0.484	−0.042
MDK	midkine (neurite growth-promoting factor 2)	0.296	0.513	−0.211
MUC1	mucin 1, cell surface associated	−0.117	0.557	−0.264
PRDM2	PR domain containing 2, with ZNF domain	−0.333	−0.012	−0.189
PXN	paxillin	0.175	0.417	0.042
SLIT2	slit homolog 2 (Drosophila)	−0.261	−0.447	0.39
**P4 Unique** **Symbol**	**Entrez Gene Name**	**P4 3 h** **Log Ratio**	**P4 12 h** **Log Ratio**	**RU486 12 h** **Log Ratio**
CTNNA1	catenin (cadherin-associated protein), alpha 1, 102 kDa	−0.175	−0.265	−0.042
ERBB3	v-erb-b2 erythroblastic leukemia viral oncogene homolog 3 (avian)	−0.069	−0.264	−0.273
FGFR2	fibroblast growth factor receptor 2	0.125	−0.545	−0.49
IGFBP5	insulin-like growth factor binding protein 5	−0.265	−0.562	0.069
IKBKB	inhibitor of kappa light polypeptide gene enhancer in B-cells, kinase beta	0.228	0.448	0.298
IL6ST	interleukin 6 signal transducer (gp130, oncostatin M receptor)	0.176	−0.014	−0.114
KCNMA1	potassium large conductance calcium-activated channel, subfamily M, alpha member 1	0.966	0.465	−0.42
NOTCH3	notch 3	−0.384	−0.559	−0.065
S100A4	S100 calcium binding protein A4	0.09	−0.785	0.031
STAT3	signal transducer and activator of transcription 3 (acute-phase response factor)	−0.223	−0.003	0.065
TCF7L2	transcription factor 7-like 2 (T-cell specific, HMG-box)	−0.207	−0.473	−0.072
TGFB1	transforming growth factor, beta 1	0.47	1.189	0.548
TGFBR3	transforming growth factor, beta receptor III	0.504	0.336	0.308

Expression changes are provided in logarithmic scale calculated as following: log (Expression treated/Expression non-treated).

Similarly, among P4 responsive genes, potential biomarkers were identified that were related to the development of the endometrium or early pregnancy: *CTNNA1, ERBB3, FGFR2, IGFBP5, IKBKB, IL6ST, KCNMA1, NOTCH3, S100A4, STAT3, TCF7L2, TGFB1,* and *TGFBR3* (Table 3).

### A Comparison of the Significant E2 and P4 Responsive Genes Identified in the Ishikawa Cell Line with a Human Endometrial Transcriptome

**Figure 1:**
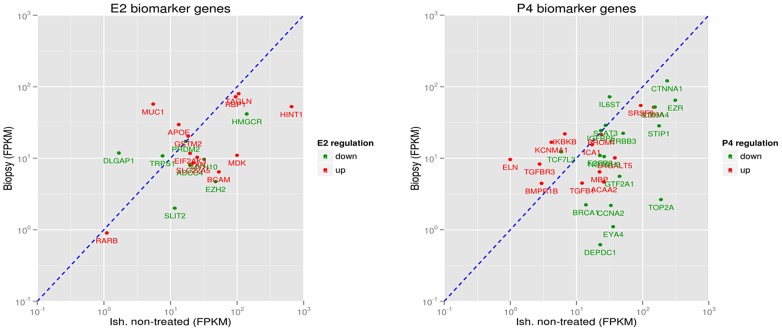
Selection of endometrial specific biomarkers found in 12 h E2 (left) and P4 (right) treated Ishikawa cells and their relative abundance in human endometrial biopsy samples at the time of embryo implantation (n = 2). Red genes up-regulated in E2 and P4 treated Ishikawa cells compared to non-treated cells; green genes down-regulated. Genes situated on the left side of the diagonal line show higher relative abundance (FPKM) in human endometrial biopsy sample compared to non-treated Ishikawa cells. Genes situated on the right side of the diagonal line show lower relative abundance (FPKM) in human endometrial biopsy sample compared to non-treated Ishikawa cells.

To predict whether the E2 and P4 responsive genes identified in Ishikawa cells were also expressed in a human endometrium, and/or have roles in the implantation process for embryos, expression of these genes were assayed in two human endometrial tissue samples collected from mid-secretory endometrium. According to RNA-Seq data, 1671 (98.8%) of E2 responsive genes, and 1668 (98.6%) of P4 responsive genes, identified in the Ishikawa cell line were also found to be expressed in human endometrium samples obtained from two patients that underwent endometrial biopsy. These human endometrium samples were only used for comparative purposes. In Table 1, the expressional abundances (e.g., FPKMs) of E2 and P4 biomarkers detected in human endometrial biopsy samples are compared with non-treated Ishikawa cells. Among the IPA-identified E2 and P4 biomarkers present in Ishikawa cells, *EZH2, MDK, MUC1, SLIT2,* and *IL6ST* were also found to be present in human endometrial transcriptomes (Figure 1).

### TAM Responsive Genes in the Ishikawa Cell Line that are Related to Reproductive System Diseases

**Figure 2:**
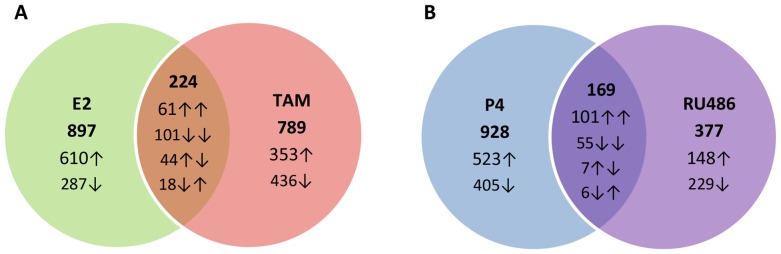
Venn diagram showing significant gene expression changes 12 h E2, TAM, P4 or RU486 treatment, relative to non-treated Ishikawa cells. A Unique and common genes after 12 h E2 and TAM treatment. B Unique and common genes after 12 h P4 and RU486 treatment. The numbers given within each of the circles represent the number of significantly changed genes unique to treatment, and arrows show the manner they are regulated (up- or down-regulation compared to non-treated Ishikawa cells). Overlaps indicate the number of commonly changed genes.

Following the treatment of Ishikawa cells with TAM for 12 h, the expression of 1013 genes were found to be significantly changed compared to non-treated Ishikawa cells. Of these genes, 432 were up-regulated and 581 were down-regulated (Table S5). These results demonstrate that TAM has both antagonistic and agonistic activity towards E2 in Ishikawa cells. Moreover, in addition to influencing E2 regulated genes, TAM significantly altered the expression of 789 genes independently from E2 (Figure 2A).

Using an IPA core analysis, the predicted function of TAM responsive genes was obtained. A total of 168 genes were found to be related to different reproductive system diseases, including uterine, ovarian, and cervical cancers, as well as genital tumors, amenorrhea, metrorrhagia, and polycystic ovary syndrome (Table 4).

**Table 4:** Selection of TAM and RU486 regulated gene products in Ishikawa cells related to reproductive system diseases.

Selection of TAM regulated gene products related to reproductive system diseases		
FunctionsAnnotation	p-Value	Molecules
adenomyosis	2,12E-05	AIG1,ANXA2,CBX6,CXXC5,DST,IQGAP1,LDHA,MALAT1,MTHFD2,TCF4,THBS1,TSPAN12
genital tumor	4,90E-05	ABCB1,ABR,ALDH3A1,ALPP/ALPPL2,ANTXR1,ANXA2,AR,ASS1,ATP1A1,BMPR1B,C9orf5,CCND1,CD44,CDH1,CDH2,CLU,COL18A1,CXXC5,ECT2,EGFL7,EHF,ENO1,EP300,EPHA2,ERBB3,ETV1,FGFR1,FGFR2,FHL2,FN1,GPC1,GPRC5A,GSTP1,HDAC4,HDAC6,HSP90AA1,ING4,ITGB4,JAG2,KIF1B,KRT23,KRT7,LDHA,LETM1,LRP5,LRRN4,MAPK8,MECOM,MKI67,MTHFD2,MYC,NCOR2,NTRK2,PAX8,PDE11A,PGR,PLEKHB1,PRC1,PSMD4,PTAFR,RACGAP1,S100A2,SAT1,SLC12A6,SLC16A3,SLC2A1,SLIT2,SMC4,SNAP25,SORT1,SRSF5,STIP1,TCF4,TFPI2,TMPRSS2,TOP2A,TRADD,TUBA1A,TUBE1,TUSC3,WT1,XIAP,ZNF217
gonadal tumor	1,66E-04	ABCB1,ALDH3A1,ALPP/ALPPL2,AR,CD44,CDH1,CLU,COL18A1,CXXC5,ECT2,ENO1,EP300,EPHA2,ERBB3,FGFR1,FGFR2,FN1,GSTP1,HDAC4,HDAC6,HSP90AA1,JAG2,KRT23,LDHA,LETM1,LRRN4,MECOM,MKI67,MTHFD2,PAX8,PGR,PTAFR,RACGAP1,S100A2,SLC12A6,SLC16A3,SLIT2,SMC4,SORT1,SRSF5,STIP1,TFPI2, TOP2A,TUBA1A,TUBE1,WT1,ZNF217
ovarian cancer	1,94E-04	ABCB1,ALDH3A1,AR,CD44,CDH1,CLU,COL18A1,CXXC5,ECT2,ENO1,EP300,EPHA2,ERBB3,FGFR1,FGFR2,FN1,GSTP1,HDAC4,HDAC6,HSP90AA1,JAG2,KRT23,LETM1,LRRN4,MECOM,MTHFD2,PAX8,PGR,PTAFR,RACGAP1,S100A2,SLC12A6,SLC16A3,SLIT2,SMC4,SORT1,SRSF5,STIP1,TFPI2,TOP2A,TUBA1A,TUBE1,WT1, ZNF217
gynecologicaldisorder	2,20E-04	ABCB1,AIG1,ALDH3A1,ANXA1,ANXA2,AR,ARL4D,ATRX,CBX6,CD44,CDH1,CDH2,CEP70,CLU,COL18A1,CTSF,CXXC5,DST,ECT2,ENO1,EP300,EPHA2,ERBB3,FGFR1,FGFR2,FN1,FOXM1,GLIPR1,GNG11,GSTP1,HDAC4,HDAC6,HSP90AA1,HSPB1,IGFBP5,IGFBP7,IQGAP1,ITGB8,JAG2,JUP,KIAA0664,KRT23,LDHA,LETM1,LRRN4,LTBP1,LTBP4,MALAT1,MECOM,MKI67,MR1,MTHFD2,MYC,NEK2,OLFM1,PAX8,PGR,PLD3,POLG,PTAFR,RACGAP1,RAD51B,RAPGEF3,S100A2,S100A4,SLC12A6,SLC16A3,SLIT2,SMC4,SORT1,SRSF5,STIP1,TAGLN,TCF4,TFPI2,TGFBR3,THBS1,TMSB10/TMSB4X,TOP2A,TPM2,TSPAN12,TUBA1A,TUBE1,WT1,ZNF217,ZNF350
cervical cancer	1,91E-03	ANXA1,ANXA2,CDH1,CDH2,CTSF,ENO1,FGFR1,FGFR2,FOXM1,GSTP1,HSP90AA1,HSPB1,ITGB8,JUP,LETM1,MKI67,PGR,SLC12A6,TAGLN,TMSB10/TMSB4X,TOP2A,TPM2,TUBA1A,TUBE1,ZNF350
uterine cancer	1,03E-02	AIG1,ANXA1,ANXA2,AR,ARL4D,CDH1,CDH2,CTSF,DST,ENO1,FGFR1,FGFR2,FOXM1,GLIPR1,GNG11,GSTP1,HSP90AA1,HSPB1,IGFBP5,IGFBP7,IQGAP1,ITGB8,JUP,KIAA0664,LETM1,LTBP1,LTBP4,MALAT1,MKI67,MR1,MTHFD2,MYC,OLFM1,PGR,PLD3,RAD51B,RAPGEF3,S100A4,SLC12A6,TAGLN,TMSB10/TMSB4X,TOP2A, TPM2,TSPAN12,TUBA1A,TUBE1,WT1,ZNF350
amenorrhea	1,30E-02	AR,PGR,TGFBR3
metrorrhagia	1,53E-02	AR,PGR
serous ovariancarcinoma	3,78E-02	CLU,CXXC5,JAG2,LRRN4,PGR,PTAFR,RACGAP1,S100A2,SLIT2,SMC4,SORT1,TFPI2
disorder of ovary	3,90E-02	AR,ATRX,CEP70,ECT2,NEK2,PGR,POLG,TGFBR3
polycystic ovarysyndrome	4,90E-02	AR,ATRX,CEP70,ECT2,NEK2,PGR
**Selection of RU486 regulated gene products related to reproductive system diseases**		
gynecologicaldisorder	5,96E-04	AGR2,ALDH3A1,AR,ARL4D,AURKB,C18orf1,CALCRL,CBX6,CDC7,CDH2,CDKN2A,CTNNAL1,CTNND1,EME1,ENO1,EPCAM,ERBB3,FGFR2,FN1,H2AFX,HDAC4,HDAC7,HDAC9,IQGAP1,JUP,KAT2B,KIAA0664,KRT23,LDHA,MALAT1,MCAM,MECOM,MYC,OLFM1,PAX8,PCM1,PDK4,PGR,RAD51B,RASSF9,RNF144B,SLC16A3,SYNC,TAGLN,TAX1BP1,TCF4,THBS1,TMSB10/TMSB4X,ZDHHC17,ZNF138,ZNF350
adenomyosis	8,84E-04	CBX6,IQGAP1,LDHA,MALAT1,SYNC,TCF4,THBS1
gonadal tumor	3,65E-03	AGR2,ALDH3A1,ALPP/ALPPL2,AR,CDKN2A,CTNNAL1,ENO1,EPCAM,ERBB3,FGFR2,FN1,H2AFX,HDAC4,HDAC7,HDAC9,KAT2B,KRT23,LDHA,MCAM,MECOM,PAX8,PGR,RASSF9,RNF144B,SLC16A3,ZNF138
metrorrhagia	4,64E-03	AR,PGR
uterine leiomyoma	4,74E-03	ARL4D,AURKB,C18orf1,CALCRL,CDC7,CTNND1,IQGAP1,KIAA0664,MALAT1,MYC,OLFM1,PDK4,PGR,RAD51B,ZDHHC17
ovarian cancer	5,10E-03	AGR2,ALDH3A1,AR,CDKN2A,CTNNAL1,ENO1,EPCAM,ERBB3,FGFR2,FN1,H2AFX,HDAC4,HDAC7,HDAC9, KAT2B,KRT23,MCAM,MECOM,PAX8,PGR,RASSF9,RNF144B, SLC16A3, ZNF138
polycystic ovarysyndrome	1,45E-02	AR,EME1,PCM1,PGR,TAX1BP1
genital tumor	1,77E-02	AGR2,ALDH3A1,ALPP/ALPPL2,AR,ARG2,AURKB,CDH2,CDKN2A,CGN,CTNNAL1,EHF,ENO1,EPCAM,ERBB3,FGFR2,FHL2,FN1,H2AFX,HDAC4,HDAC7,HDAC9,KAT2B,KRT23,LDHA,MCAM,MECOM,MTA1,MYC,NCOR2,NTRK2,OAZ1,PAX8,PGR,RASSF9,RCAN2,RNF144B,SLC16A3,TCF4,TMPRSS2,ULK3,ZNF138
atypical endometrialhyperplasia	2,84E-02	PGR
metastasis of cervicalcancer cell lines	2,84E-02	ZNF350
preterm birth	2,84E-02	PGR
primaryhypogonadism	2,84E-02	AR
subfertility	2,84E-02	PGR
serous ovariancarcinoma process	3,07E-02	CTNNAL1,EPCAM,H2AFX,MCAM,PGR,RASSF9,RNF144B,ZNF138
amenorrhea	3,11E-02	AR,PGR
*The categories related to male infertility and breast cancer are excluded*		

**Figure 3:**
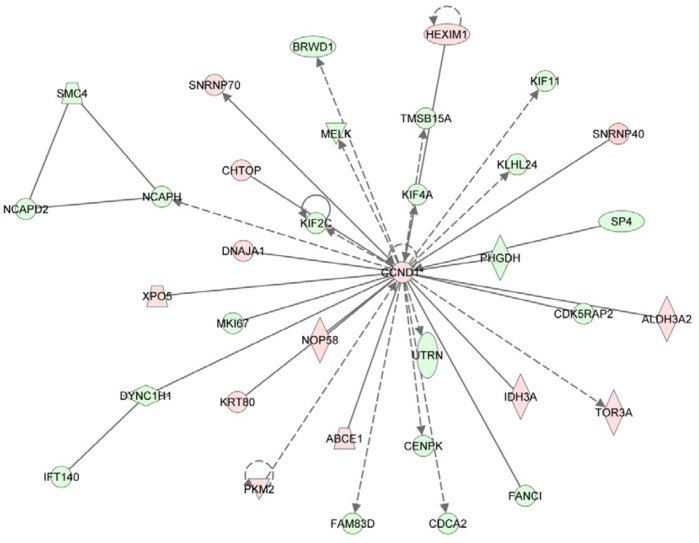
Top 1 network with TAM 12 h significant genes related to DNA replication, recombination and repair, cell cycle, cellular assembly and organization. Red molecules represent up-regulated and green down-regulated genes among TAM 12 h significant genes in Ishikawa cells. The networks were generated through the use of IPA (Ingenuity® Systems, www.ingenuity.com).

One of the main signalling pathways identified from TAM responsive genes was associated with regulation of DNA replication, recombination, and repair, as well as cell cycle progression, and cellular assembly and organization. Moreover, TAM responsive genes encoded molecules that directly, or indirectly, were associated with the cell cycle regulator, cyclin D1 (*CCND1*). Correspondingly, *CCND1* was found to be significantly up-regulated 12 h after treatment with TAM (Figure 3).

### RU486 Significant Genes in the Ishikawa Cell Line are Related to Reproductive System Diseases

Treatment of Ishikawa cells with the P4 antagonist, RU486, for 12 h resulted in significant changes in the expression of 546 genes compared with untreated cells, with 255 genes up-regulated and 291 genes down-regulated (Table S6). Similar to TAM, RU486 exhibits both agonistic and antagonistic activities for P4 in Ishikawa cells. For example, 377 genes responsive to RU486 after 12 h did not show significant changes in expression after treatment with P4 for 12 h. Therefore, these genes are regulated independently by RU486 and in the absence of P4 in Ishikawa cells (Figure 2B).

**Figure 4:**
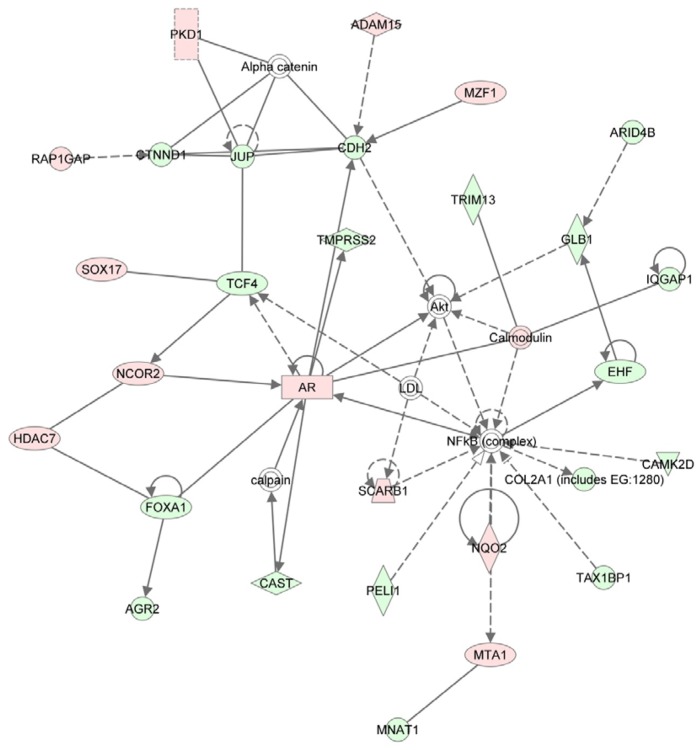
Top one network formed among RU486 significant genes related to gene expression, cell-to-cell signalling and interaction, and tissue development. The central molecules in the network were cadherin 2 (CDH2), AR and NFκB complex. The networks were generated through the use of IPA (Ingenuity® Systems, www.ingenuity.com).

Of the 546 genes found to be responsive to treatment with RU486, 86 encoded molecules related to diseases of the reproductive system. For example, molecules related to adenomyosis, gonadal tumours, metrorrhagia, and uterine leiomyoma were identified (Table 4). Based on the genes that were responsive to treatment with RU486, a signalling pathway related to gene expression, cell-to-cell signalling and interactions, and tissue development was identified. The central molecules in this network, including cadherin 2 (CDH2) and a complex between AR and NFκB, mediate direct and indirect interactions with RU486 responsive genes (Figure 4). For example, the transcriptional co-repressor gene, *NCOR2*, was up-regulated as was the androgen receptor (*AR*) gene. However, transcription factor, FOXA1, was down-regulated. Genes associated with the cell to cell contact and adhesion were also down-regulated, and included: *CTNND1, JUP, CDH2, IQGAP1,* and *COL2A1.* Alternatively, *PDK1* and *ADAM15* were up-regulated, and have roles in the induction of tissue breakdown. Most of the molecules in this network were also related to cell death and apoptosis.

## Discussion

The aim of this study was to define the transcriptional response of an endometrial model to treatment with E2, P4, TAM, and RU486. To our knowledge, this is the first report of the application of RNA-Seq to the study of early genome-wide effects in a human endometrial cell line. Moreover, the majority of genes that were found to be significantly responsive to E2 and P4 in the Ishikawa cell line were also detected in human endometrial biopsies collected at embryo implantation.

During the last decade, microarrays have been the most commonly used method for performing endometrial transcriptome analyses in order to identify genes differentially expressed in proliferative and secretory phases. However, the genes identified in these various studies have not been consistent. These differences could be due to variations in the study design used, and/or other limiting factors of microarray analysis. The prominent limitations associated with microarray analyses include hybridization and cross-hybridization artefacts, differences in data analysis, and low and variable coverage of all genes present in commercially available array platforms [30]. RNA-Seq is a method that has very low, if any, background signal since DNA sequences can be mapped to unique regions of the genome [31]. In addition, unlike DNA microarrays, RNA-Seq does not have any upper limit for quantification, thereby facilitating the detection of genes expressed at very low or very high levels.

Various genes were found to have very high mRNA expression levels following treatment of cells with E2 or P4. These included *PSAP, ATP5G2*, *ATP5H*, and *GNB2L1*, which all showed highly abundant transcripts in response to E2 or P4. Housekeeping genes were also highly expressed in all experiments. In previous work, glycoprotein coding gene *PSAP* has been shown to be up-regulated in the endometrium during GnRH antagonist-treated cycles [7]. This protein has been shown to participate in lysosomal hydrolysis of sphingolipids [32]. *PSAP* also has a predicted estrogen response element (ERE) site in its promoter region [33]. The data from the present study are consistent with these previous results, with high levels of *PSAP* detected 12 h after E2 treatment. The ATP synthase subunits, *ATP5G2* and *ATP5H*, as well as the cell proliferation-inducing gene, *GNB2L1*, were found to be highly expressed following treatments with E2 and P4. Although these three genes have not been described in relation to the endometrium in earlier studies, their high levels of expression in response to steroid hormones could be crucial for achieving a receptive state in the endometrium, a tissue which undergoes rapid developmental changes on a monthly basis.

Using Cufflinks and an IPA biomarker filter, genes found to be significantly affected by E2 and P4 were also genes that have been previously characterized as important to the functioning of the endometrium. For example, the expression of histone methyltransferase (*EZH2*) was found to be down-regulated 12 h after E2 treatment compared to non-treated cells, yet the expression was remained 12 h after TAM treatment. Moreover, loss of EZH2 activity in the endometrium has been shown to contribute to the epigenetic programming of decidualizing endometrial stromal cells [34]. An increase in level of *MUC1* mRNA was detected 12 h after treatment with E2 compared to non-treated Ishikawa cells. *MUC1* mRNA was also detected in P4- and RU486-treated Ishikawa cells, although the increases observed were not statistically significant. In the latter case, this may be due to the short duration of the P4 treatment, and different results may be obtained if longer hormonal treatments were used. However, data regarding the role of MUC1 continues to be conflicting as some investigators report an increase in MUC1 in the endometrium during the receptive phase of the endometrium [35], [36], while others have reported the opposite result, including the disappearance of MUC1 from pinopodes [37], [38]. *MUC1* expression was also found to be not solely dependent on P4 receptors but most likely mediated via non-genomic pathways [39].

Paxillin plays an important role in cellular cytoskeletal formation, and the gene for paxillin (*PXN*) was found to undergo significant up-regulation 12 h after treatment with E2. While treatment with P4 also slightly increased the abundance of *PXN*, the increase was not significant. During the endometrial decidualization process, PXN has been shown to participate in integrin-mediated signal transduction pathways [40]. In these pathways, fibroblast growth factor receptor 2 (*FGFR2*) also has a role, and is expressed in the endometrium at the beginning of the secretory phase. This expression profile coincides with the development of endometrial oedema and the formation of a complex, subepithelial capillary plexus [41]. Based on the RNA-Seq microarray data of the present study, *FGFR2* was relatively abundant in non-treated and E2-treated cells. However, its expression significantly decreased after treatment with P4 for 12 h. Insulin-like growth factor binding protein-5 (*IGFBP5*) was also significantly down-regulated after treatment with P4 for 12 h. This is consistent with a previous report that P4 inhibits the expression of *IGFBP5* during embryo implantation [42]. Furthermore, 98.8% and 98.6% of the genes in Ishikawa cells that were significantly up-regulated following treatment with E2 and P4, respectively, were also present in the human endometrial transcriptome during embryo implantation.

Significant changes in expression were not detected for several known endometrial biomarkers in this study. This may be due to the *in vitro* conditions assayed, or the duration of hormone and modulator treatments applied. For example, expression of leukaemia inhibitory factor (LIF) was not detected, which has previously been shown to be important during embryo implantation in both animal and human studies [43]. Moreover, expression of LIF should be the highest in the luminal and glandular epithelium during the luteal phase [44]. However, the *in vitro* conditions assayed in the present study did not imitate the physiological conditions of the luteal phase since E2 and P4 were applied separately. Expression of the receptor for LIF, LIFR, was detected, although it did not undergo significant changes in expression following hormone/antagonist treatments. On the other hand, expression of *IL6ST*, a co-receptor of LIFR, was significantly up-regulated after 3 h of P4 treatment. Interestingly, another well-known endometrial marker, vascular endothelial growth factor (VEGF), was not identified in our dataset, although *VEGFB* showed relatively high abundance in all experiments and was slightly (albeit not significantly) up-regulated after 12 h of P4 treatment. Based on these results, and those of a previous study that performed immunostaining of VEGF in both glandular epithelial cells and stromal cells during the mid-secretory phase of the human endometrium [45], epithelial Ishikawa cells may not be the best model for studying endometrial vascularization and *VEGF* expression. In addition, expression of epidermal growth factor (EGF) was relatively low, and did not change significantly following any treatment. Given that the endometrium is a complex tissue with different tissue components being important for signal transduction, epithelial cells alone do not sufficiently represent these complexities. In addition, during the natural cycles, endometrial tissue is longer exposed to ovarian steroid hormones. Therefore, some of the effects observed when using these tissues could be induced by stromal factors. In addition, the changes in mRNA expression reported in the present study represent early changes that occurred following the administration of E2, P4, and their respective modulator treatments. Therefore, it is still possible that these changes could activate the cascade of molecular changes that are eventually needed to achieve a receptive endometrium and successful embryo implantation in humans.

An additional objective of this study was to examine the molecular effects of TAM and RU486 on Ishikawa cells. TAM transduces its signal by competing with E2 for receptor binding, and exhibits antagonistic, or agonistic, activity towards E2 in a tissue-specific manner [46], [47]. Moreover, it has been well-documented that the use of TAM is associated with a 2-to-7-fold increase in the incidence of endometrial cancer in TAM-treated patients [48]. However, the molecular mechanisms responsible for the endometrial aberrations observed remain unclear. In a previous study that compared the estrogenic effect of TAM and E2 in Ishikawa cells, the reported gene expression profile was highly diverse and ligand specific. As a result, it was hypothesized that TAM influences the transcriptional response of a specific subset of genes in the uterus [49]. In another study where the endometrial cell line, ECC-1, was treated with TAM versus E2, it was also observed that TAM regulates a subset of specific genes distinct from E2. This result was further confirmed *in vivo* when 256 genes were specifically identified in TAM-treated patients [50], [51]. In the current study, 1013 genes were observed to undergo significant changes in expression after 12 h of TAM treatment. Moreover, only 224 of these genes overlapped with genes that underwent changes following treatment with E2 for 12 h. In addition, a total of 168 of these TAM-specific genes were associated with various diseases of the reproductive system. Consistent with *in vivo* data obtained from postmenopausal women treated with TAM [50], [51]), cyclin D1 (*CCND1*) was found to be significantly up-regulated 12 h after treatment with TAM in the Ishikawa cell line. CCND1 was also found to be a central molecule in a signalling network identified from the TAM responsive genes revealed in the current study. This network was associated with mediating early processes related to DNA replication, recombination and repair, as well as cell cycle progression, cellular assembly, and cellular organization. Amplification or overexpression of *CCND1* has been shown to play a pivotal role in the development of several human cancers, including parathyroid adenoma, breast cancer, colon cancer, lymphoma, melanoma, and prostate cancer [52]. In addition, several studies have been reported an abundance of CCND1 in endometrial carcinoma [53], [54], which is hypothesized to be caused by altered protein degradation and nuclear export due to mutations present in threonine 286 of the *CCND1* coding region [55].

In most cases, TAM exhibited antagonistic activity towards E2 specific genes. However, a subset of genes was found to be similarly regulated (e.g., up-regulated or down-regulated) after both E2 and TAM treatments, thereby demonstrating agonistic activity. Specifically, TAM and E2 both up-regulated mRNAs of *AR, FGFR1, KIAA0664, MALAT1, OLFM1, TMSB10/TMSB4X, TPM2, JAG2, PAX8,* and *SRSF5.* Moreover, this set of genes is believed to be related to uterine, ovarian, and cervical cancers [56], [57]. In addition, RNA-Seq data from the present study indicated that the mRNA of ferritin light chain (*FTL*) was present in very high abundance after 12 h of E2 treatment and TAM treatment. Ferritin is the major intracellular iron storage protein in cells and variations in ferritin subunit composition has the potential to affect the rates of iron uptake and release in different tissues [58].

The classical P4 antagonist, RU486, has been used for emergency contraception and the medical termination of pregnancies up to 49 days after gestation based on its ability to block the action of P4 by binding to its receptor expressed by the endometrium. As a result, impaired endometrial maturation leads to degeneration and shedding of the endometrial lining, thereby preventing or disrupting implantation of the conceptus [59], [60]. In women, a single dose of mifepristone (200 mg) during the secretory phase of a cycle rapidly renders the endometrium unreceptive, and has been shown to alter gene expression in the uterus within 6 h of oral administration [61], [62]. When considering these effects on the endometrium, it is also important to consider that RU486 has both antagonistic and agonistic activities towards PRs, yet exhibits additionally anti-glucocorticoid and anti-androgenic activities [63]. The results of the RNA-Seq analysis in the present study found that proline dehydrogenase 1 (*PRODH*) was down-regulated and thrombospondin 1 (*THBS1*) was up-regulated following RU486 administration, and these results are consistent with those described for the human endometrium [64]. In addition, the genes, follistatine-like 1 (*FSTL1*) and epidermal growth factor receptor (*ERBB3*), showed similar levels of down-regulation after 12 h of treatment with RU486, and these results are consistent with those previously reported for rhesus monkeys in response to treatment with RU486 for four days [65]. Previous studies have also shown that treatment with RU486 in the early luteal phase inhibits normal down-regulation of the PR gene (*PGR*) [66]. Correspondingly, expression of *PGR* was found to be significantly up-regulated after 12 h of treatment with RU486, in our analysis. However, the observation that *PGR* expression was also up-regulated after 12 h of P4 treatment is inconsistent with previous studies. Most likely, longer P4 treatments are needed for *PGR* suppression.

A functional analysis of RU486 responsive genes in Ishikawa cells identified a signalling pathway associated with cell-to-cell signalling, cell-to-cell interactions, and tissue development. Central molecules of this signalling pathway include CDH2, AR, and NFκB. The early molecular effects of RU486 also appear to involve the down-regulation of adhesion molecules and the induction of molecules related to apoptosis. For example, calcium-dependent cadherin CDH2, which is responsible for cell-cell adhesion, was found to be significantly suppressed following treatment with RU486. Similar was seen for adhesion molecule JUP. Nuclear receptor AR and nuclear co-repressor, NCOR2, were both found to be up-regulated following RU486 treatment. Correspondingly, in a previous study their recruitment was shown to be enhanced by RU486 [67]. While the expression of NFκB did not directly change, it was directly, or indirectly, linked to molecules related to apoptosis. As a result, it could be responsible for endometrial tissue shedding and early abortions of the conceptus.

In conclusion, these results provide valuable insight into the mechanisms of early steroid hormone signalling and the consequences of antagonist/agonist action in the human endometrium. However, studies of other *in vitro* models, as well as an analysis of additional human samples, is needed to confirm the early endometrial changes observed to be mediated by E2 and P4 and their modulators in this study.

## Supporting Information

Figure S1 E2 significant genes in Ishikawa cell line. 1691 known genes showed significantly changed mRNA expression after 3 h (first column) and 12 h (second column) E2 treatment. 12 h TAM treatment (third column) had antagonistic activity on most of the E2 significant genes instead of 61 genes, which showed similar up-regulated expression and 101 genes, which had similar down-regulative expression pattern after E2 and TAM treatments. For data visualization hierarchical clustering was used. Genes were clustered by taking account E2 significant genes after 3 h and 12 h treatment and compared to 12 h TAM.
(TIF)

Figure S2 P4 significant genes in Ishikawa cell line. The expression of 1692 known genes was significantly changed after 3 h (first column) and 12 h (second column) P4 treatment. 12 h RU486 treatment (third column) had antagonistic activity on most of the P4 significant genes instead of 101 genes, which showed similar up-regulated expression and 55 genes, which had similar down-regulated expression pattern after P4 and RU486 treatments.
(TIF)

Figure S3 Genes up (red) – or down (green) regulated by ERα (ESR1) in Ishikawa cells after 12 h E2 treatment. The networks were generated through the use of IPA (Ingenuity® Systems, www.ingenuity.com).
(TIF)

Figure S4 Genes up (red) – or down (green) regulated by PRA (PGR) in Ishikawa cells after 12 h P4 treatment. The networks were generated through the use of IPA (Ingenuity® Systems, www.ingenuity.com).
(TIF)

Table S1 FPKM values of genes in active transcriptome of non-treated, E2, P4, TAM and RU486 treated Ishikawa cells and human endometrial biospy samples (n = 2). (XLSX)

Table S2 Gene expression changes significantly changed after E2 treatment in Ishikawa cell line compared to non-treated cells. (XLSX)

Table S3 Gene expressions significantly changed after P4 treatments of Ishikawa cells. (XLSX)

Table S4 Biomarkers related to reproductive system diseases or endocrine system disorders among E2 and P4 significant genes in Ishikawa cell line. (XLS)

Table S5 Gene expressions (lndiff) significantly changed after TAM treatment in Ishikawa cells. (XLSX)

Table S6 Gene expressions (lndiff) significantly changed after RU486 treatment in Ishikawa cells. (XLSX)
