# Supplementary figures and images for: Changes in the Transcriptome of the Human Endometrial Ishikawa Cancer Cell Line Induced by Estrogen, Progesterone, Tamoxifen, and Mifepristone (RU486) as Detected by RNA-Sequencing
Source: PLoS One. 2013 Jul 16;8(7):e68907. doi: 10.1371/journal.pone.0068907 (PMC3712916; doi:10.1371/journal.pone.0068907)

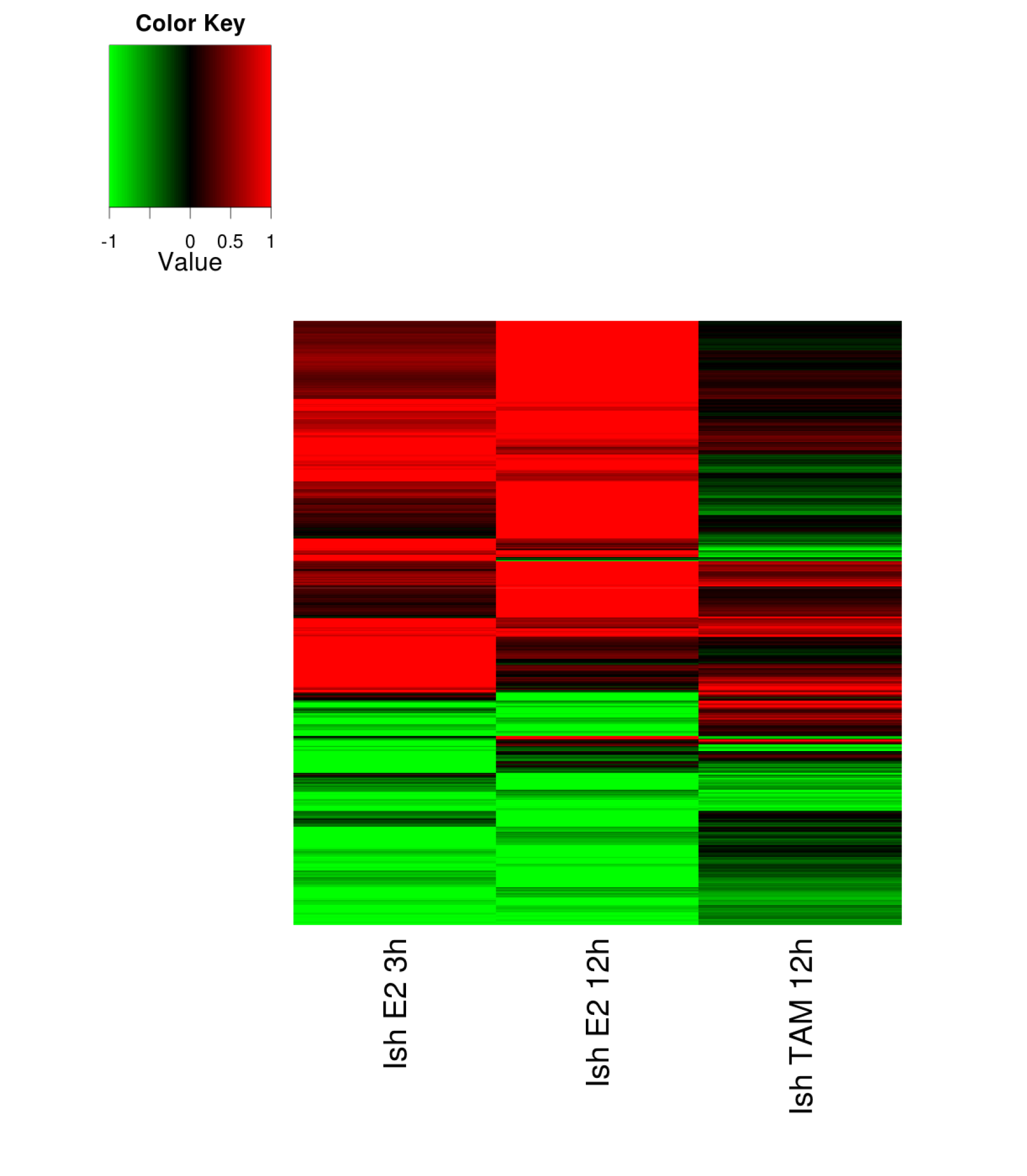

Supplement: Figure S1 — 1691 known genes showed significantly changed mRNA expression after 3 h (first column) and 12 h (second column) E2 treatment. 12 h TAM treatment (third column) had antagonistic activity on most of the E2 significant genes instead of 61 genes, which showed similar up-regulated expression and 101 genes, which had similar down-regulative expression pattern after E2 and TAM treatments. For data visualization hierarchical clustering was used. Genes were clustered by taking account E2 significant genes after 3 h and 12 h treatment and compared to 12 h TAM. (TIF) [file pone.0068907.s001.tif]

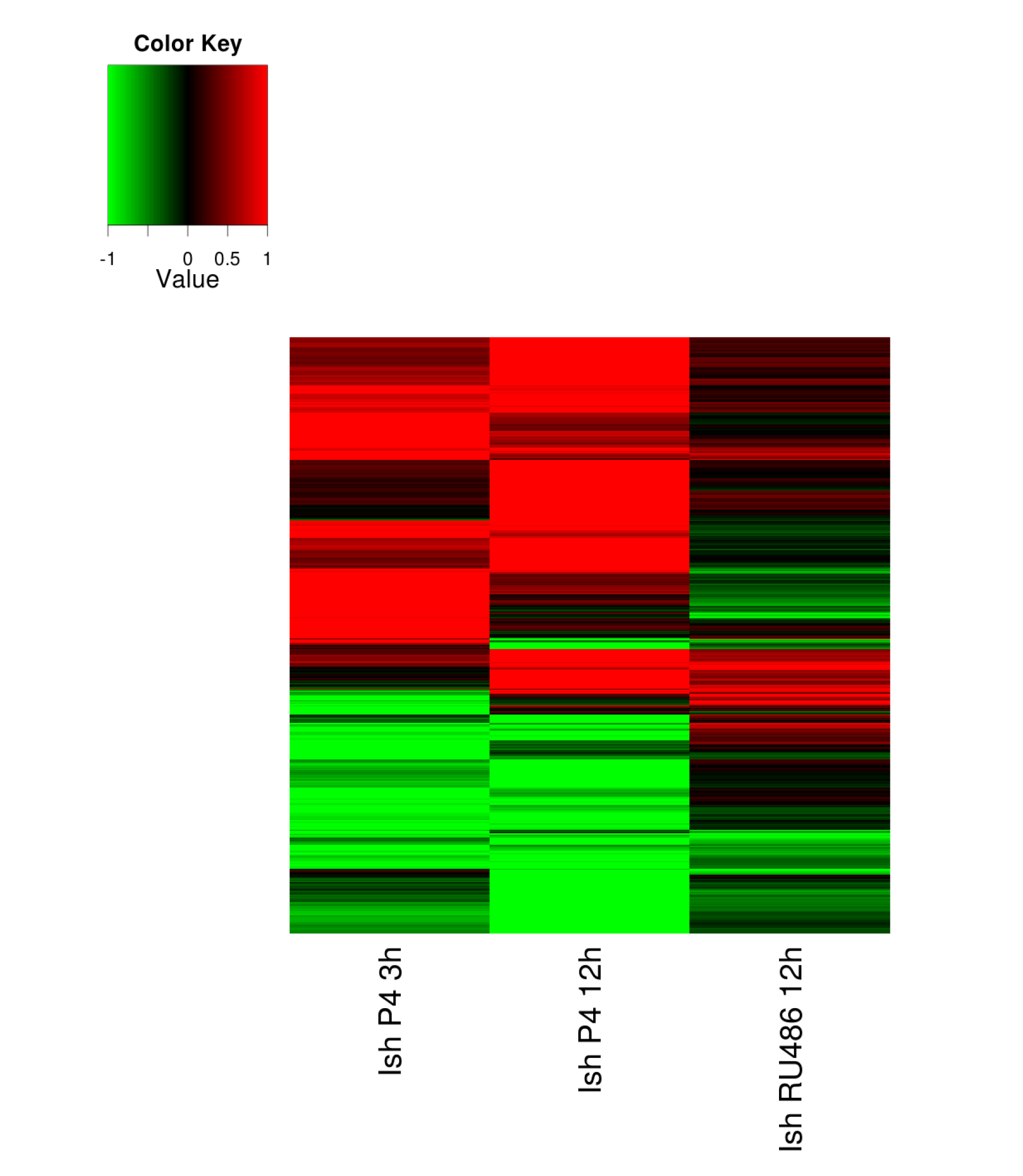

Supplement: Figure S2 — The expression of 1692 known genes was significantly changed after 3 h (first column) and 12 h (second column) P4 treatment. 12 h RU486 treatment (third column) had antagonistic activity on most of the P4 significant genes instead of 101 genes, which showed similar up-regulated expression and 55 genes, which had similar down-regulated expression pattern after P4 and RU486 treatments. (TIF) [file pone.0068907.s002.tif]

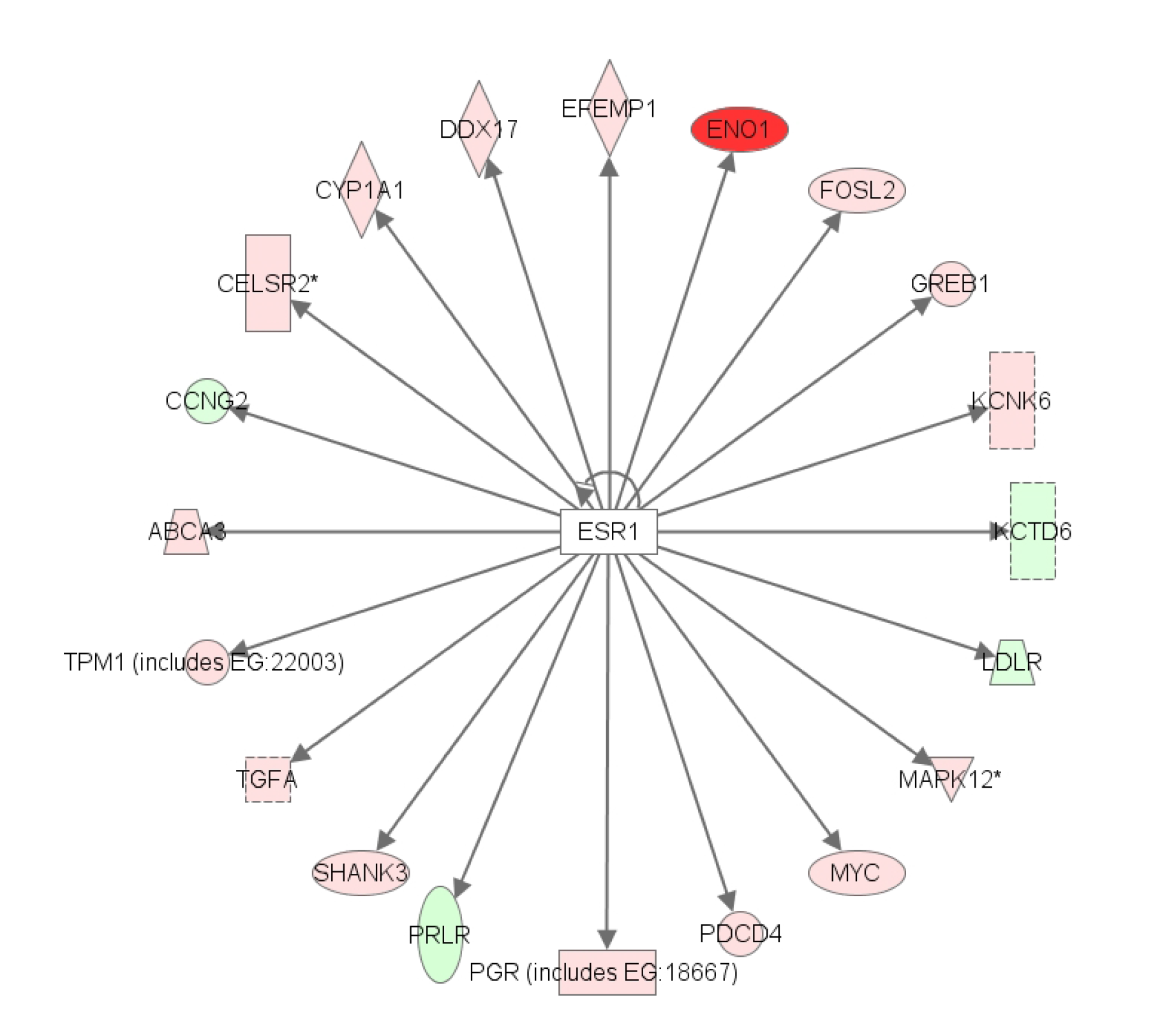

Supplement: Figure S3 — The networks were generated through the use of IPA (Ingenuity® Systems, www.ingenuity.com). (TIF) [file pone.0068907.s003.tif]

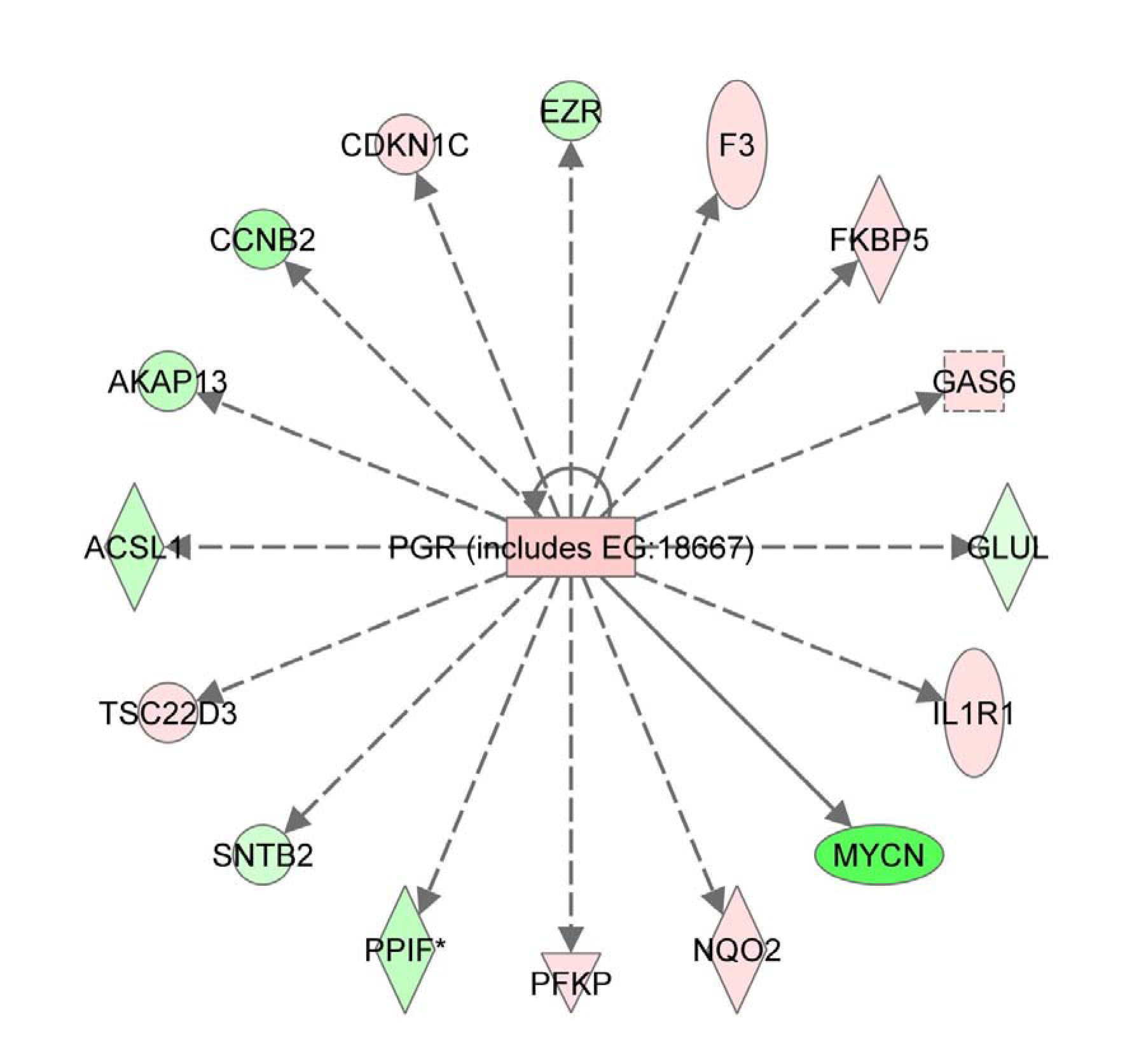

Supplement: Figure S4 — The networks were generated through the use of IPA (Ingenuity® Systems, www.ingenuity.com). (TIF) [file pone.0068907.s004.tif]
